# Exploring the Association of Religion and Religiosity on Mammography Screening Among White and Black Women: Results from the Brazilian Longitudinal Study of Aging (Elsi-Brazil)

**DOI:** 10.1007/s10943-026-02694-3

**Published:** 2026-06-12

**Authors:** Claudio Santiago Dias, Ana Paula Verona, Victor Leocádio

**Affiliations:** 1 Department of Sociology, Universidade Federal de Minas Gerais (UFMG), Avenida Antonio Carlos, 6627, Belo Horizonte, Minas Gerais 31270-901, Brazil; 2 Department of Demography, Center for Development and Regional Planning (CEDEPLAR), Universidade Federal de Minas Gerais (UFMG), Belo Horizonte, Minas Gerais Brazil; 3 Center for Development and Regional Planning (CEDEPLAR), Universidade Federal de Minas Gerais (UFMG), Belo Horizonte, Minas Gerais Brazil; 4 Federal Institute for Population Research (BiB), Wiesbaden, Germany

**Keywords:** Breast cancer, Mammography, Religious denomination, Religious attendance, Racial inequalities, Brazil

## Abstract

This study examines the relationship between religious denomination, religious attendance, and mammography screening among White and Black women in Brazil, using baseline data from the Brazilian Longitudinal Study of Aging (ELSI-Brazil, 2015–2016). The sample comprised 3120 women aged 50–69 who self-identified as White or Black (including Brown). Mammography use was categorized as never, within the past two years (in accordance with national guidelines), or more than two years ago. Multinomial logistic regression models assessed associations, controlled by sociodemographic, and health-related factors. Results show that Evangelical women were significantly more likely than Catholics to have never undergone a mammogram (*RRR* = 1.53) and to be overdue (*RRR* = 1.52). Higher religious attendance was associated with a lower risk of never having been screened. These findings underscore a dual role of religion: it can either facilitate preventive care through, for instance, social support and health-promoting norms, or hinder it when certain beliefs—such as “faith healing”—discourage medical engagement. Furthermore, racial disparities persist within Evangelicals. Among Black women, Evangelical affiliation was associated with a higher likelihood of never being screened, while high religious attendance substantially reduced this risk. Among White women, Evangelical affiliation did not influence initiation of screening but was linked to nearly double the risk of being overdue, with no significant effect of attendance. Ultimately, our findings underscore the complex intersection of religion, race, and health. This study makes a novel contribution by being the first in Brazil to assess how religious denomination and religiosity relate to mammography screening among racial groups. Future research on religion and health should account for racial disparities.

## Introduction

The World Health Organization (WHO) has shown that cancer is one of the leading causes of death worldwide (Sung et al., 2021). According to the International Agency for Research on Cancer (IARC), an estimated twenty million new cancer cases were documented globally in 2022. Of these, 11.6% were related to breast cancer. During the same period, 9.7 million cancer-related deaths were recorded, with 6.9% caused by breast cancer (Bray et al., 2024). In Brazil, this is the second most common type of cancer among women and the leading cause of cancer-related death in this population, with 73,000 new cases each year (Santos et al., 2023).

Several studies indicate that mammography screening is one of the main strategies used for the early detection of breast cancer in asymptomatic women, alongside the early diagnosis of cases presenting suspicious signs and symptoms (Barbosa et al., 2019; Migowski et al., 2018; Rodrigues et al., 2021). Women enrolled in health programs that regularly perform mammography exams are more likely to have smaller tumors and are less likely to require more invasive surgeries. Furthermore, they show a more favorable response to available treatments compared to those who do not undergo mammography at the recommended frequency (Fancellu et al., 2019; James et al., 2016; Rodrigues et al., 2021; Tabár et al., 2019).

This study extends understanding of women’s health, religion, and race/ethnicity in Brazil by examining the association between religious denomination, religious attendance, and mammography exams, comparing two racial groups: White and Black (both black and mixed-race). The analysis focuses on women aged 50 to 69, utilizing data from the 2015/2016 Brazilian Longitudinal Study of Aging.

This study is guided by three research questions: (1) Is there an association between religious denomination, religiosity, and the frequency of mammography screening in Brazil? (2) Are there differences based on race/ethnicity in the frequency of these screenings? (3) How do the associations between religious denomination and religiosity with the frequency of mammography screenings vary within and across racial groups?

Several studies in various contexts and countries have examined the relationship between religion and the demand for preventive health services (Kretzler et al., 2022). However, this study is the first to analyze, within the Brazilian context, the association between religious denomination, religiosity, and the frequency of mammography screenings among racial groups in the country.

### Religion, Race and Health

The Brazilian Ministry of Health recommends that women aged 50 to 69 undergo mammography at least every two years. This recommendation is based on scientific evidence showing that this age group benefits the most from a significant reduction in preventable deaths that could occur if breast cancer is not detected early. For women with a family history of breast cancer, the recommendation is to begin mammography screening at a younger age (Urban et al., 2017).

Access to mammography in Brazil still reflects racial, socioeconomic, and regional inequalities. In general, access is greater in the southern and southeastern regions of the country, among White women, those with higher income and education levels, and those who report better self-perceived health (Amorim et al., 2008; Barbosa et al., 2019; Oliveira et al., 2011; Ramos et al., 2018; Schäfer et al., 2021).

With regarding to racial inequalities, empirical evidence indicates that Black and Brown women are at a disadvantage compared to White women in accessing this service in Brazil. Data from the National Health Survey (2019) showed that 61.8% of White women aged 50 to 69 had undergone a mammography within two years before the interview. Among Brwon and Black women, this percentage was 54.4% and 56.5%, respectively (Amorim et al., 2008; Oliveira et al., 2011; Schäfer et al., 2021).

Unlike race, women's religious denomination and religiosity have been relatively underexplored in studies on mammography practice in Brazil. The lack of focus on these factors is notable, given that research across different countries and contexts has consistently found a strong association between religion and health. (Ringdal 1986; Ellison & Levin, 1998; Verona et al. 2011; Koenig et al., 2012; Cartwright, 2021; Verona et al., 2024).

Religion can positively influence the health of its practitioners through different mechanisms. One such mechanism involves guidance and encouragement toward healthy habits (such as abstinence from alcohol and tobacco). Some religions, for example, emphasize that the body is "the temple of the Holy Spirit" and thus should be well cared for (Cartwright, 2021; Regnerus & Smith, 2005). Another mechanism is the support networks. These networks can foster social interactions and reciprocity, aiding in both preventive and general health care for church members and helping them cope with adversities such as illness (Santos et al. 2011), family conflicts, and financial difficulties (Ellison & Levin, 1998; Mariz, 1994).

However, there are also explanations for the negative association eventually found between religion and health. This relationship may occur if a particular religious denomination questions medical practices and the advancement of scientific treatment and disease prevention (Fournet et al., 2018). In this context, it is essential to be aware of the rise of Evangelical Pentecostalism in Brazil, driven by an emphasis on "*faith healing*." For the Christian faith, this is a gift of the Holy Spirit through which practitioners can heal from physical, mental, and social illnesses without medical intervention. Previous studies suggest that "*faith healing*" may be associated with a reduced demand for medical and preventive treatments and even an increased risk of mortality among members of different religious groups (Bartkowski et al., 2011).

## Material and Methods

### ELSI-Brazil Database

The data used in this work were retrieved from the ELSI-Brazil baseline survey. This cohort study is representative of the Brazilian population aged 50 and older who are not institutionalized (Lima-Costa et al., 2018). The baseline study was conducted from May 2015 to October 2016, collecting information from 9412 individuals residing in 70 municipalities across Brazil's five macro-regions and in 21 states plus the Federal District (Lima-Costa et al., 2018).

### Study Population

This study used information from women aged 50 to 69, initially totaling 3831 observations. Only women who self-identified as White, Black, or Brwon were included in the analysis, excluding others such as indigenous and oriental origins (Japanese, Chinese, and Korean, among others) (N = 108). Regarding the variable of religious denomination, women who identified as having no religion were excluded (N = 114), as religious attendance was not asked for them. In addition to these exclusions, women who did not respond to questions about race/ethnicity, religious denomination, frequency of mammography, education, and self-assessed health were removed from the sample. The final sample size was 3120 women.

### Dependent Variable (Frequency of Mammography Screening)

The following question was considered to analyze the frequency of mammography screening among women: "When was the last time you had a mammography or breast X-ray?" The original response categories were: (1) never, (2) less than one year ago, (3) one year to less than two years ago, (4) two years to less than three years ago, (5) three years or more.

These categories were regrouped into three groups as follows:

- Women who have never had a mammography (original category 1).
- Women who had a mammography less than two years ago (original categories 2 and 3).
- Women who had a mammography more than two years ago (original categories 4 and 5).

This new categorization was performed to allow for the comparison of three distinct groups of women regarding mammography frequency: those who have never had the exam, those who have had it within the past two years (in line with the Ministry of Health's recommendation), and those who are overdue for the exam (Moreira et al., 2023; Oliveira et al., 2011).

### Independent Variables (Religious Denomination and Religiosity)

The questionnaire includes the following question about religious denomination: "What is your religion?" The response categories are: (1) none, (2) Catholic, (3) Protestant, (4) Evangelical, (5) Spiritist/Kardecist, (6) Buddhist, (7) Muslim, (8) African-derived religions, (9) others, (99) not specified/no response. Religious denomination was recategorized as follows: (1) Catholic, (2) Evangelicals (Protestants and Pentecostals), and (3) other religions (all other categories). It is important to mention that the “other religions” category should not be interpreted, as it aggregates all remaining reported religions and therefore constitutes a highly heterogeneous group. For this reason, no specific interpretations were made for the results of this category, although it was retained in the analysis to avoid additional exclusions from the sample.

Religiosity, measured by religious attendance, was recategorized as follows: high frequency (attends religious services more than once a week), moderate frequency (once a week or two to three times a month), and low frequency (once or a few times a year or never).

Because the analyses were stratified by race/skin color, we created a categorical variable from responses to the question: “Which of the following options best describes your skin color?” The options were: (1) White, (2) Black, (3) Brown, (4) Oriental (e.g., Japanese, Chinese, or Korean), (5) Indigenous, and (9) Not specified/No response. Only women who self-identified as White, Black, or Brown were included in this study. For racial comparisons, respondents identifying as Brown were combined with those identifying as Black to form a single “Black” category. Although Black and Brown women are distinct groups, they experience similar disadvantages relative to White women in mammography screening uptake (Schafer et al., 2021). Moreover, the number of women reporting as Black alone was too small to analyze separately. The final analytic sample therefore comprised 1213 White women (38.8%) and 1907 Black women (62.2%). The merging of the “Black” and “Brown” categories into a single category is common in Brazilian health disparities research. For example, Hone et al. (2017) adopted this strategy in a national mortality study to improve comparability and reduce potential inconsistencies in racial classification. Tomasiello et al. (2024) likewise combined self-declared Black and Brown individuals into a single category to examine racial and income inequalities in access to healthcare in Brazilian cities.

### Control Variables

The control variables used were: (1) Age: Continuous variable ranging from 50 to 69 years; (2) Marital status: single, married or cohabitating, divorced or separated, and widowed.; (3) Education level: no schooling, incomplete or complete elementary education, incomplete or complete secondary education, and incomplete or complete higher education; (4) Health insurance: yes or no; (5) Self-perceived health: continuous variable ranging from 1 to 5 (with 1 being very good or excellent, 2 good, 3 fair, 4 poor, and 5 very poor); (6)Tobacco use: yes or no; (7) Geographic region: South, Southeast, Central-West, North, and Northeast; and (8) Place of residence: rural or urban.

### Data Analysis

Descriptive analyses were performed by computing relative frequencies to profile the study population and to estimate the prevalence of the outcome—mammography utilization—both overall and within each racial group.

We examined the association between mammography frequency and both religious denomination and frequency of religious practice using multinomial logistic regression, reporting relative risk ratios (RRRs) with 95% confidence intervals. Statistical significance was defined as *p* < 0.05. The ELSI-Brazil study's sampling design (Lima-Costa et al., 2018) was considered in the analyses. The R version 4.0.2 was used for all statistical computations.

### Ethics Committee

The ELSI-Brazil study received ethical approval from the Research Ethics Committee of the René Rachou Institute, Oswaldo Cruz Foundation (Fiocruz), Rio de Janeiro, Brazil, and is registered on Plataforma Brasil (protocol No. 886,754).

## Results

### Descriptive Results

Table 1 summarizes mammography‐screening status among Black women aged 50–69, stratified by religious variables, and demographic and sociodemographic characteristics. Overall, 19.9% had never undergone a mammogram, 60.1% were up to date (screened within the past two years), and 20.0% were overdue. Evangelical women—who comprised 34.1% of the sample—showed less favorable screening patterns: 21.2% had never undergone a mammogram and only 57.6% were up to date. By contrast, among Catholic women 61.0% were current, and 20.0% had never been screened.

**Table 1:** Mammography screening history and religion and sociodemographic characteristics among black women *Source*: ELSI-Brazil, 2015/2016

Variables	%	Last time you had a mammography		
		Never	Less than two years	Two years or more
*Religious denomination*				
Catholic	61.3	20.0	61.0	19.0
Evangelical	34.1	21.2	57.6	21.2
Other Religions ^1^	4.6	9.7	66.0	24.3
*Religious frequency*				
Low	8.5	29.0	47.4	23.6
Medium	39.4	19.4	61.1	19.6
High	52.1	18.9	61.3	19.8
*Age* (mean)	58.0	58.8	57.7	58.3
*Marital status*				
Single	16.4	22.9	58.5	18.7
Married or cohabitating	56.8	18.6	62.4	18.9
Divorced or separated	13.7	18.2	59.8	22.0
Widow	13.2	23.7	52.1	24.2
*Level of education*				
No education	13.1	39.7	40.5	19.8
Incomplete or complete elementary education	59.0	20.5	59.1	20.4
Incomplete or complete high school	20.2	9.5	69.1	21.4
Incomplete or complete higher education	7.7	8.8	77.4	13.8
*Health insurance*				
No	81.8	22.7	56.6	20.7
Yes	18.2	7.3	75.6	17.1
*Self-perceived health* (mean)	2.73	2.73	2.70	2.83
*Tobacco use*				
No	16.9	18.3	62.3	19.4
Yes	83.1	27.7	49.2	23.1
*Geographic region*				
North	8.5	34.4	48.5	17.0
Northeast	34.0	23.2	61.0	15.8
Southeast	42.7	13.2	63.2	23.6
South	7.0	19.6	59.3	21.1
Central-West	7.8	27.3	51.9	20.8
*Residence location*				
Rural	17.7	35.0	49.4	15.6
Urban	82.3	16.7	62.4	21.0
*Total (%)*	100.0	19.9	60.1	20.0
*Total (N)*	1907	414	1102	391

1—The “other religions” category should not be interpreted because it is a highly heterogeneous group

Table 1 highlights a clear gradient by religious attendance. Women who attended services infrequently had the highest proportion who had never been screened (29.0%) and the lowest proportion who were up-to-date (47.4%). By contrast, women with frequent attendance were more likely to follow screening guidelines, with 61.3% current on their mammograms.

Regarding the control variables, mean age did not differ significantly across groups. Marital status, however, revealed clear disparities: married or cohabiting women had the highest prevalence of recent screening (62.4%), whereas widowed and single women were less likely to comply, showing higher proportions of overdue or no mammography. Educational attainment emerged as one of the strongest predictors of screening behavior. Among women with no formal schooling, nearly 40% had never undergone a mammogram and only 40.5% were up to date. By contrast, 77.4% of women with higher education had been screened recently, and only 8.8% had never had the exam.

In addition, health-insurance coverage proved to be a powerful enabling factor: 75.6% of insured women were up to date with screening, compared with just 56.6% of those without insurance. Self-rated health did not differ significantly across screening categories. Tobacco use, however, showed a clear negative association—27.7% of smokers had never undergone a mammogram, versus 18.3% of non-smokers.

Geographic disparities were also evident. Women living in the Southeast showed the highest screening rate (63.2%), whereas those in the North had the greatest proportion who had never been screened (34.4%). Rural residence was likewise associated with poorer outcomes: 35.0% of rural women had never undergone a mammogram and only 49.4% were up to date—figures that contrast sharply with urban women, 62.4% of whom were current on screening and just 16.7% had never been screened.

Table 2 presents mammography-screening behavior among 1213 White women aged 50–69, stratified by religious variables, and demographic and sociodemographic characteristics. Relative to Black women, White women demonstrated higher adherence: only 10.1% had never undergone a mammogram, 68.0% were up to date (screened within the past two years), and 21.9% were overdue.

**Table 2:** Mammography screening history and religion and sociodemographic characteristics among white women *Source:* ELSI-Brazil, 2015/2016

Variables	%	Last time you had a mammography		
		Never	Less than two years	Two years or more
*Religious denomination*				
Catholic	68.0	10.8	69.6	19.6
Evangelical	22.3	11.9	56.4	31.8
Other Religions ^1^	9.7	1.2	83.1	15.7
*Religious frequency*				
Low	13.1	9.7	66.7	23.7
Medium	46.1	10.1	69.3	20.6
High	40.8	10.3	66.9	22.8
*Age* (mean)	58.3	58.5	58.2	58.4
*Marital status*				
Single	10.9	20.0	52.4	27.6
Married or cohabitating	64.6	8.5	72.0	19.5
Divorced or separated	13.1	9.2	62.4	28.4
Widow	11.4	11.1	66.3	22.6
*Level of education*				
No education	4.4	30.3	51.1	18.5
Incomplete or complete elementary education	57.8	12.0	62.7	25.3
Incomplete or complete high school	23.3	5.8	76.4	17.8
Incomplete or complete higher education	14.5	3.6	80.3	16.1
*Health insurance*				
No	32.3	13.5	62.0	24.5
Yes	67.7	2.9	80.6	16.5
*Self-perceived health* (mean)	2.5	2.4	2.5	2.8
*Tobacco use*				
No	83.9	8.8	70.4	20.9
Yes	16.1	17.2	55.4	27.4
*Geographic region*				
North	2.1	39.9	43.6	16.5
Northeast	11.6	23.2	59.8	17.1
Southeast	54.4	8.0	69.4	22.6
South	25.8	6.4	72.1	21.5
Central-West	6.0	9.1	62.2	28.7
*Residence location*				
Rural	10.0	21.9	53.9	24.2
Urban	90.0	8.8	69.5	21.7
*Total (%)*	100.0	10.1	68.0	21.9
*Total (N)*	1213	137	808	268

1—The “other religions” category should not be interpreted because it is a highly heterogeneous group

Religious denomination was strongly associated with screening outcomes. Among White women, Evangelicals—22.3% of the sample—showed lower adherence, with only 56.4% having been screened in the past two years and 31.8% reporting overdue exams. In contrast, 69.6% of Catholic women were up to date, and only 10.8% had never been screened.

Unlike the pattern observed among Black women, religious attendance among White women showed weaker associations with screening behavior. Non-screening rates were consistent across levels of attendance (ranging from 9.7 to 10.3%), and the proportion of women up to date with screening ranged only modestly—from 66.7 (low attendance) to 69.3% (medium attendance). This suggests that religiosity may play a less prominent role in shaping screening behavior in this subgroup.

Age was uniformly distributed across screening categories, with no meaningful variation. In contrast, marital status demonstrated a clear pattern: women who were married or cohabiting reported the highest screening adherence (72.0%), while single women had the lowest (52.4%). Single women also had the highest proportion of non-screening (20.0%) and overdue exams (27.6%).

Educational level had a substantial impact. Among women with no formal education, nearly one-third (30.3%) had never undergone a mammogram, and only 51.1% were up to date. These figures improved progressively with higher educational attainment. White women with higher education had the best indicators: 80.3% had undergone screening within the past two years, and only 3.6% had never done so.

Health insurance coverage was another key factor associated with better outcomes. Among insured women—who made up 67.7% of the sample—80.6% were up to date, and only 2.9% had never been screened. In contrast, uninsured women had lower screening coverage (62.0%) and higher rates of non-screening (13.5%) and overdue exams (24.5%).

Self-perceived health was not a major distinguishing variable, although women who had never undergone screening reported slightly better average health (2.4) than those overdue (2.8). Tobacco use, however, showed a clear negative association: 17.2% of smokers had never had a mammogram, and 27.4% were overdue, compared to 8.8% and 20.9%, respectively, among non-smokers.

Geographic region and urban vs. rural residence revealed substantial disparities. White Women in the South and Southeast exhibited the highest adherence to screening guidelines (72.1% and 69.4%, respectively). The North region presented the highest proportion of women who had never undergone a mammogram (39.9%), despite comprising a small portion of the sample (2.1%). Women living in urban areas were significantly more likely to be up to date (69.5%) than those in rural settings (53.9%). Additionally, rural women had more than twice the rate of non-screening (21.9%) compared to their urban counterparts (8.8%).

### Regression Models

Table 3 presents the results of the multinomial logistic regression model assessing the association between religious denomination, religious attendance, race, and the frequency of mammography screening among all women aged 50 to 69. As shown in Panel A, compared to Catholic women, Evangelical women had a significantly higher relative risk of never having undergone a mammogram (*RRR* = 1.53; *p* = 0.018) and a higher relative risk having had it more than two years ago (*RRR* = 1.52; *p* = 0.003). These findings suggest a consistent pattern in which Evangelical affiliation is associated with lower adherence to recommended mammography screening, regardless of religious frequency and sociodemographic factors.

**Table 3:** Multivariate logistic regression analysis of religious and sociodemographic factors influencing mammography screening among all women. ELSI-Brazil 2015/2016 *Source*: ELSI-Brazil. 2015/2016

Variables	Panel A				Panel B			
	Never				Two years or more			
	RRR	P value	95% CI		RRR	P value	95% CI	
*Religious Denomination*								
Catholic								
Evangelical	1.53	0.018*	1.077	2.175	1.52	0.003*	1.155	2.007
Other Religions ^1^	0.47	0.119	0.178	1.219	0.92	0.663	0.621	1.355
*Religious frequency*								
Low								
Medium	0.61	0.003*	0.442	0.843	0.77	0.127	0.554	1.076
High	0.52	0.001***	0.353	0.752	0.71	0.075	0.492	1.035
*Age*	1.03	0.010**	1.007	1.049	1.02	0.072	0.999	1.035
*Marital Status*								
Single								
Married or cohabitating	0.53	0.003*	0.346	0.807	0.74	0.058	0.541	1.011
Divorced or separated	0.60	0.009**	0.409	0.879	1.01	0.960	0.690	1.478
Widow	0.64	0.031*	0.426	0.961	0.95	0.781	0.662	1.363
*Level of Education*								
No education								
Incomplete or complete elementary education	0.44	0.000***	0.291	0.653	0.85	0.450	0.566	1.288
Incomplete or complete high school	0.21	0.000***	0.131	0.328	0.76	0.210	0.494	1.168
Incomplete or complete higher education	0.20	0.000***	0.096	0.409	0.65	0.047*	0.419	0.995
*Health Insurance*								
No								
Yes	0.33	0.000***	0.213	0.496	0.63	0.001***	0.482	0.814
*Self-perceived health*	0.82	0.007**	0.710	0.946	1.25	0.001***	1.090	1.429
*Tobacco use*								
No								
Yes	2.45	0.000***	1.685	3.575	1.56	0.008**	1.123	2.171
*Geographic region*								
North								
Northeast	0.30	0.002*	0.139	0.647	0.69	0.111	0.431	1.091
Southeast	0.22	0.000***	0.122	0.387	1.06	0.755	0.738	1.518
South	0.17	0.000***	0.081	0.365	0.90	0.637	0.564	1.420
Central-West	0.48	0.026*	0.255	0.916	1.19	0.394	0.795	1.788
*Residence location*								
Rural								
Urban	0.41	0.000***	0.271	0.606	0.79	0.125	0.580	1.069
*Race*								
Black								
White	0.78	0.115	0.573	1.063	1.11	0.376	0.880	1.402

P < 0.05*, P < 0.01**, P < 0.001***(two-tailed tests)

1—The “other religions” category should not be interpreted because it is a highly heterogeneous group

Religious attendance, on the other hand, was positively associated with screening behavior. Women with high religious attendance had a 48% lower relative risk of never having had a mammogram (*RRR* = 0.52; *p* = 0.001), and those with moderate attendance also showed a significant protective effect (*RRR* = 0.61; *p* = 0.003), compared to women with low religious attendance. Although the associations between religious attendance and being overdue for screening (more than two years ago) did not reach statistical significance, the relative risks for both high (*RRR* = 0.71; *p* = 0.075) and moderate attendance (*RRR* = 0.77; *p* = 0.127) indicated a protective trend (Panel B, Table 3).

Regarding racial differences, White women had a lower relative risk of never having had a mammogram compared to Black women (*RRR* = 0.78; *p* = 0.115), although this result was not statistically significant at the 5% level. Interestingly, when considering women who were overdue for mammography, White women had a higher risk than Black women (*RRR* = 1.11; *p* = 0.376), but again the difference was not statistically significant.

Among control variables, women who were or had ever been in a union presented a lower relative risk of never having had mammography (compared to having had one in the past two years) compared to single women. This risk was also lower among women with higher levels of education and those living in urban areas of the country.

Table 4 presents the results of the multinomial logistic regression model examining the association between religious denomination, religious attendance, and the frequency of mammography screening among Black women. The findings reveal that religion significantly influence whether women undergo mammography as recommended. As shown in Panel A of Table 4, compared to Catholic women, Evangelical women had a 54% higher relative risk of never having undergone a mammogram (*RRR* = 1.54; *p* = 0.056), a result that, although marginally significant, suggests a potential religious barrier to preventive health behavior in racial this group. In contrast, religious attendance appears to have a protective effect. Black women with high levels of religious attendance had a 65% lower relative risk of never having had a mammogram (*RRR* = 0.35; *p* < 0.001) than to those with low attendance. These results suggest that regular engagement in religious activities may facilitate access to or encourage the uptake of preventive health services among Black women.

**Table 4:** Multivariate logistic regression analysis of religious and sociodemographic factors influencing mammography screening among black women. ELSI-Brazil 2015/2016 *Source*: ELSI-Brazil. 2015/2016

Variables	Panel A				Panel B			
	Never				Two years or more			
	RRR	P value	95% CI		RRR	P value	95% CI	
*Religious denomination*								
Catholic								
Evangelical	1,54	0,056	0,989	2,388	1,28	0,271	0,826	1,969
Other Religions ^1^	0,84	0,778	0,252	2,809	1,27	0,408	0,722	2,225
*Religious frequency*								
Low								
Medium	0,42	0,002*	0,246	0,716	0,69	0,199	0,388	1,219
High	0,35	0,000***	0,195	0,627	0,62	0,159	0,324	1,205
*Age*	1,03	0,024*	1,004	1,061	1,02	0,104	0,996	1,040
*Marital status*								
Single								
Married or cohabitating	0,71	0,154	0,443	1,138	0,96	0,807	0,687	1,340
Divorced or separated	0,68	0,094	0,434	1,068	1,11	0,698	0,665	1,837
Widow	0,89	0,586	0,594	1,343	1,32	0,228	0,839	2,083
*Level of education*								
No education								
Incomplete or complete elementary education	0,43	0,000***	0,273	0,684	0,72	0,219	0,420	1,221
Incomplete or complete high school	0,19	0,000***	0,117	0,323	0,70	0,210	0,404	1,222
Incomplete or complete higher education	0,21	0,000***	0,098	0,459	0,48	0,020*	0,263	0,889
*Health insurance*								
No								
Yes	0,36	0,000***	0,210	0,609	0,64	0,022*	0,441	0,939
*Self-perceived health*	0,86	0,077	0,726	1,017	1,13	0,151	0,956	1,342
*Tobacco use*								
No								
Yes	2,24	0,001***	1,404	3,565	1,43	0,094	0,940	2,175
*Geographic region*								
North								
Northeast	0,29	0,006**	0,122	0,698	0,66	0,095	0,407	1,076
Southeast	0,23	0,000***	0,113	0,451	1,01	0,950	0,725	1,408
South	0,29	0,005**	0,126	0,685	0,93	0,801	0,541	1,607
Central-West	0,63	0,195	0,318	1,264	1,07	0,773	0,666	1,728
*Residence location*								
Rural								
Urban	0,41	0,000***	0,263	0,636	0,93	0,714	0,636	1,365

P < 0.05*, P < 0.01**, P < 0.001***(two-tailed tests)

1—The “other religions” category should not be interpreted because it is a highly heterogeneous group

Interestingly, for the outcome of being overdue for a mammogram (more than two years ago), neither religious denomination nor attendance was significantly associated with increased or decreased risk, although the direction of the estimates followed a similar pattern (Panel B, Table 4).

Table 5 presents the results of the multinomial logistic regression model examining the association between religious denomination, religious attendance, and the frequency of mammography screening among White. The findings indicate that Evangelical women were no more likely than Catholic women to have never undergone a mammogram. (*RRR* = 1.48; *p* = 0.163) (Panel A, Table 5). However, when examining the likelihood of being overdue for the exam—defined as having had a mammogram more than two years ago—White Evangelical women showed a substantially higher relative risk (*RRR* = 1.98; *p* < 0.001) (Panel B, Table 5).

**Table 5:** Multivariate logistic regression analysis of religious and sociodemographic factors influencing mammography screening among white women. ELSI-Brazil 2015/2016 *Source*: ELSI-Brazil. 2015/2016

Variables	Panel A				Panel B			
	Never				Two years or more			
	RRR	P value	95% CI		RRR	P value	95% CI	
*Religious denomination*								
Catholic								
Evangelical	1,48	0,163	0,851	2,586	1,98	0,000***	1,393	2,815
Other Religions ^1^	0,12	0,004**	0,028	0,505	0,73	0,249	0,432	1,245
*Religious frequency*								
Low								
Medium	1,08	0,812	0,559	2,098	0,84	0,391	0,553	1,262
High	0,85	0,686	0,394	1,847	0,78	0,198	0,534	1,140
*Age*	1,02	0,325	0,978	1,068	1,01	0,387	0,983	1,045
*Marital status*								
Single								
Married or cohabitating	0,24	0,000***	0,119	0,484	0,44	0,005**	0,249	0,779
Divorced or separated	0,42	0,025*	0,195	0,894	0,73	0,265	0,422	1,269
Widow	0,24	0,001***	0,102	0,548	0,50	0,041*	0,260	0,971
*Level of education*								
No education								
Incomplete or complete elementary education	0,50	0,053	0,244	1,009	1,42	0,399	0,627	3,223
Incomplete or complete high school	0,23	0,002**	0,088	0,579	1,09	0,850	0,428	2,797
Incomplete or complete higher education	0,14	0,019*	0,027	0,718	1,20	0,698	0,479	3,000
*Health insurance*								
No								
Yes	0,27	0,003**	0,115	0,635	0,62	0,025*	0,406	0,941
*Self-perceived health*	0,69	0,008**	0,518	0,906	1,49	0,000***	1,215	1,816
*Tobacco use*								
No								
Yes	2,93	0,001***	1,558	5,495	1,76	0,011*	1,141	2,721
*Geographic region*								
North								
Northeast	0,28	0,007**	0,109	0,700	0,92	0,914	0,188	4,474
Southeast	0,12	0,000***	0,052	0,294	1,29	0,750	0,271	6,113
South	0,07	0,000***	0,026	0,184	1,02	0,980	0,211	4,939
Central-West	0,12	0,001***	0,036	0,425	1,62	0,539	0,345	7,625
*Residence location*								
Rural								
Urban	0,40	0,009**	0,199	0,793	0,61	0,019*	0,400	0,921

P < 0.05*, P < 0.01**, P < 0.001*** (two-tailed tests)

1—The “other religions” category should not be interpreted because it is a highly heterogeneous group

Religious attendance was not significantly associated with mammography screening behavior among White women. Neither moderate nor high frequency of religious participation showed statistically significant associations with the outcomes of never having had the exam or being overdue.

## Discussion

This study investigated whether religious affiliation and service attendance are associated with mammography‐screening frequency in Brazil, comparing White women with Black women (the latter category combining self-identified Black and Brown respondents). We analyzed data from women aged 50–69 years who participated in the baseline wave (2015–2016) of the Brazilian Longitudinal Study of Aging (ELSI-Brazil).

A substantial body of research indicates that religious involvement can enhance both well-being and health-related behaviors. Several mechanisms have been proposed. First, many faith traditions prescribe behavioral norms—such as abstaining from alcohol, tobacco, or other potentially harmful substances—that can directly promote better health (Cartwright, 2021; Ellison & Hummer, 2010; Koenig et al., 2012; Mariz, 1994; Verona et al., 2024). Second, religious communities often provide emotional and social support during periods of adversity, thereby strengthening individuals’ coping capacities (Benjamins et al., 2006; Santos et al. 2011). Finally, congregational settings nurture support networks and social capital, which have been linked to lower risks of mental-health disorders and substance misuse (Moreira-Almeida et al., 2006; Upenieks, 2020). These interpersonal ties and the collective monitoring that occurs within religious groups may discourage unhealthy behaviors and reinforce protective ones.

Yet religion can also exert adverse effects. In Brazil, the rapid expansion of Pentecostal Evangelical churches has been propelled in part by their emphasis on “faith healing”—the belief that the Holy Spirit grants divine cures for physical, mental, or social ailments, often obviating the need for medical treatment (Chesnut, 1997 and 2011; Brown, 2011). Evidence suggests that adherence to such doctrines can discourage preventive and clinical care, potentially worsening health outcomes (Cerqueira-Santos et al., 2004) and even increasing mortality risk (Bartkowski et al., 2011).

Our first research question asked whether religious denomination and service attendance are linked to mammography-screening frequency in Brazil. The analysis confirms that they are: even after adjusting for religiosity, race/skin color, and multiple demographic and socioeconomic covariates, Evangelical women aged 50–69 were less likely than Catholic women to undergo mammography. Compared with Catholic women who had been screened within the previous two years, Evangelical women aged 50–69 were 53% more likely never to have undergone a mammogram and 52% more likely to be overdue. These results suggest that Evangelical affiliation may discourage preventive behaviors, such as mammography screening—perhaps reflecting the “faith-healing” doctrines promoted in some Pentecostal congregations. However, it is important to emphasize that, although evidence of faith healing has been documented in several ethnographic studies of evangelical churches in Brazil (Chesnut, 1997, 2011), the ELSI questionnaire does not include a specific measure of this phenomenon. Consequently, the data analyzed in this article do not allow for a direct assessment of the hypothesis that the observed association between religion and mammography screening is attributable to faith healing.

Our results also indicate that religious attendance is negatively associated with the likelihood of undergoing mammography. In other words, women with medium to high levels of religious attendance—regardless of their specific religious affiliation—have a lower risk of never having had a mammogram or of being overdue for one, compared to those with low religious attendance. A possible explanation for this is that they may be more exposed to guidance, interactions, and support networks within the churches that help promote healthier life habits. Nevertheless, it is important to highlight that these results may also be attributable to reverse causality, as women who frequently participate in religious activities may exhibit better health and greater mobility—compared to those who attend infrequently or not at all—which could, in turn, positively influence their mammogram screening practices (Regnerus & Smith, 2005; Zimmer et al., 2019).

Taken together, the findings for Evangelical women and those with high religious attendance may underscore the dual role of religion—as both a potential barrier and a facilitator—in shaping mammography practices among women age 50 to 69 in Brazil. On one hand, religious affiliation and frequency may be beneficial when they provide access to health-related information and foster supportive networks that can be mobilized in times of need (Cartwright, 2021; Verona et al., 2024). On the other hand, religion may discourage preventive care and medical treatment when religious teachings promote, for instance, the belief that healing can be achieved solely through *'faith healing'* (Cerqueira-Santos et al., 2004; Bartkowski et al., 2011; Chesnut, 1997 and 2011).

The second research question of this study was: Are there differences based on race/ethnicity in the frequency of mammography screenings? Results from Table 3 showed no significant differences, when controlling by religious denomination, religiosity, and sociodemographic factors. This finding contrasts with prior empirical research, which has frequently documented racial disparities in mammography utilization, often indicating that Black women have reduced access to mammograms compared to White women (Amorim et al., 2008; Oliveira et al., 2011; Schäfer et al., 2021).

The third and final research question was: How do the associations between religious denomination and religiosity with the frequency of mammography screenings vary within and across racial groups? This question was addressed by the results of Tables 4 and 5. Examining differences within the group of Black women, Evangelical women exhibited a higher relative risk of never having undergone mammography compared to Catholic women. However, this finding should be interpreted with caution since Evangelical affiliation was associated with never having undergone mammography screening among Black women at a marginal level of statistical significance (p = 0.056). Because the association does not reach the conventional 5% threshold for statistical significance, it should be regarded as suggestive rather than conclusive.

Another important finding is that, among Black women, the likelihood of never having had a mammogram was lower for those with higher frequency of religious attendance, regardless of the denomination they belong to. One possible interpretation of the potential protective association between high religious attendance and the practice of mammography screening among them is that religious communities may operate as important sources of social support and health-related information, particularly in socioeconomically disadvantaged contexts in Brazil (Chesnut, 1997). Frequent participation in religious services may strengthen interpersonal ties and facilitate access to practical forms of support, such as encouragement to seek preventive care and in accessing and using health services (Ellison & Levin, 1998; Koenig et al., 2012). In addition, churches may function as spaces where information about health, prevention, and screening circulate through formal or informal networks. Thus, the observed association may reflect not only individual religiosity, but also the role of religious communities as social environments that promote preventive health behaviors (Koenig et al., 2012).

Finally, neither religious denomination nor religious attendance was significantly associated with the risk of being overdue for a mammogram among Black Women.

Among White women, neither religious affiliation nor service attendance was significantly linked to the likelihood of never having undergone a mammogram. However, White Evangelical women faced nearly double the risk of being overdue compared with White Catholic women. These results suggest that among White women, religious denomination—particularly Evangelical affiliation—plays a more prominent role than religious attendance in shaping adherence to mammography screening. While Evangelical women are not less likely to ever engage in screening than Catholic women, however, they are significantly more likely to fall behind on regular exams, pointing to possible barriers related to ongoing participation in preventive care rather than initial access.

### Limitations

This study has several limitations. First, due to its cross-sectional design, we could not determine the direct causal impact of religion and religiosity on mammography practice. For instance, healthier, more mobile women may access services more frequently, which could potentially inflate the observed effect of religiosity on screening adherence. That is, the positive association between religious attendance and health may be affected by selection processes and reverse causality (Koenig et al., 2012; Regnerus & Smith, 2005). Because attendance at religious services requires some level of physical mobility, psychological well-being, and social integration, healthier individuals may be more likely to participate regularly. Conversely, individuals with poorer health, functional limitations, or depressive symptoms may reduce their participation in religious activities. Therefore, the relationship between religious attendance and health should be interpreted cautiously, with emphasis on the observed association rather than on causal claims about the effect of religious participation on health outcomes.

Second, a better understanding of the relationship between religion and mammography practice could be achieved by including a broader range of faith traditions and non-religious belief systems and by exploring the psychosocial mechanisms that link religiosity to preventive health care. Finally, future research should investigate the individual motivations and beliefs that influence mammography decisions beyond religious and racial factors.

## Conclusion

Overall, this article yields two principal findings. First, when all women aged 50–69 are considered together, Evangelicals are more likely both to have never had a mammogram and to be overdue for the exam than Catholics. Regarding religious attendance, women who attend services frequently are less likely to have never undergone a mammogram or to be overdue. This evidence suggests that religion can both facilitate and hinder preventive health behaviors, depending on denomination and level of engagement.

Second, racial disparities exist among Evangelicals: Black Evangelical women were less likely than their Black Catholic counterparts ever to have been screened, whereas White Evangelical women were less likely than White Catholics to remain current with recommended screening intervals. Yet frequent service attendance appeared protective only among Evangelical Black women, who benefit from lower odds of never having been screened when they attend services more often.

This pioneering study in Brazil reveals that religion plays a multifaceted role in mammography screening practices among women aged 50 to 69, with effects varying by religious affiliation, frequency of service attendance, and race. Our findings indicate a paradox: while Evangelical affiliation may be associated with a lower likelihood of screening, a high frequency of church attendance appears, in some cases, to be linked to better preventive practices, possibly due to the benefits of social support. Although the study found no overall racial differences in mammography access, subgroup analyses suggest that religious factors have distinct impacts on White and Black women. Ultimately, our findings underscore the complex intersection of religion, race, and health. Future research on religion and health should account for racial disparities.
